# Myths and Common Misbeliefs About Colorectal Cancer Causation in Palestine: A National Cross-Sectional Study

**DOI:** 10.1200/GO.23.00295

**Published:** 2024-01-02

**Authors:** Mohamedraed Elshami, Shoruq Ahmed Naji, Mohammad Fuad Dwikat, Ibrahim Al-Slaibi, Mohammed Alser, Mohammed Ayyad, Balqees Mustafa Mohamad, Wejdan Sudki Isleem, Adela Shurrab, Bashar Yaghi, Yahya Ayyash Qabaja, Fatma Khader Hamdan, Raneen Raed Sweity, Remah Tayseer Jneed, Khayria Ali Assaf, Maram Elena Albandak, Mohammed Madhat Hmaid, Iyas Imad Awwad, Belal Khalil Alhabil, Marah Naser Alarda, Amani Saleh Alsattari, Moumen Sameer Aboyousef, Omar Abdallah Aljbour, Rinad AlSharif, Christy Teddy Giacaman, Ali Younis Alnaga, Ranin Mufid Abu Nemer, Nada Mahmoud Almadhoun, Sondos Mahmoud Skaik, Bettina Bottcher, Nasser Abu-El-Noor

**Affiliations:** ^1^ Division of Surgical Oncology, Department of Surgery, University Hospitals Cleveland Medical Center, Cleveland, OH; ^2^ Ministry of Health, Gaza, Palestine; ^3^ Faculty of Pharmacy, Al-Azhar University of Gaza, Gaza, Palestine; ^4^ Faculty of Medicine, An-Najah National University, Nablus, Palestine; ^5^ Almakassed Hospital, Jerusalem, Palestine; ^6^ The United Nations Relief and Works Agency for Palestine Refugees in the Near East (UNRWA), Gaza, Palestine; ^7^ Faculty of Medicine, Al-Quds University, Jerusalem, Palestine; ^8^ Doctors Without Borders (Médecins Sans Frontières), Hebron, Palestine; ^9^ Faculty of Medicine, Islamic University of Gaza, Gaza, Palestine; ^10^ Palestine Medical Complex, Khanyounis, Palestine; ^11^ Faculty of Dentistry, Arab American University, Jenin, Palestine; ^12^ Augusta Victoria Hospital, Jerusalem, Palestine; ^13^ Faculty of Allied Medical Sciences, Arab American University, Jenin, Palestine; ^14^ Faculty of Medicine, Al-Azhar University, Gaza, Palestine; ^15^ Faculty of Nursing, Islamic University of Gaza, Gaza, Palestine

## Abstract

**PURPOSE:**

To explore public awareness of myths around colorectal cancer (CRC) causation in Palestine and to examine factors associated with good awareness.

**MATERIALS AND METHODS:**

Convenience sampling was used to recruit adult Palestinians from governmental hospitals, primary health care centers, and public spaces. Recognizing 13 myths around CRC causation was assessed using a translated-into-Arabic version of the Cancer Awareness Measure-Mythical Causes Scale. Awareness level was determined based on the number of CRC mythical causes recognized: poor (0-4), fair (5-9), and good (10-13). Multivariable logistic regression was used to examine the association between sociodemographic characteristics and displaying good awareness. It adjusted for age group, sex, education, occupation, monthly income, residence, marital status, having chronic diseases, being a vegetarian, knowing someone with cancer, and site of data collection.

**RESULTS:**

Of 5,254 participants approached, 4,877 agreed to participate (response rate, 92.3%). A total of 4,623 questionnaires were included in the final analysis: 2,700 from the West Bank and Jerusalem (WBJ) and 1,923 from the Gaza Strip. Only 219 participants (4.7%) demonstrated good awareness of myths around CRC causation. WBJ participants were twice more likely than those from the Gaza Strip to display good recognition (5.9% *v* 3.1%). Male sex, living in the WBJ, and visiting hospitals were all associated with an increase in the likelihood of displaying good awareness. Conversely, knowing someone with cancer was associated with a decrease in the likelihood of displaying good awareness. Having a physical trauma was the most recognized CRC causation myth (n = 2,752, 59.5%), whereas eating food containing additives was the least (n = 456, 9.8%).

**CONCLUSION:**

Only 4.7% displayed good ability to recognize myths around CRC causation. Future educational interventions are needed to help the public distinguish the evidence-based versus mythical causes of CRC.

## Teaser

A national cross-sectional study showed very poor awareness of colorectal cancer causation myths in Palestine.

## INTRODUCTION

Worldwide, colorectal cancer (CRC) is the third most frequent cancer and the third leading cause of cancer-related deaths.^1^ In Palestine, CRC accounted for 14.3% of cancer-related deaths in 2021, making it the second most common cause of those deaths.^2^

**CONTEXT**

**Key Objective**
This study evaluated the public awareness of myths around colorectal cancer (CRC) causation in the Palestinian community and examined the sociodemographic factors associated with exhibiting good awareness.
**Knowledge Generated**
The awareness level of CRC causation myths was very poor, with only 219 participants (4.7%) demonstrating good awareness. Factors associated with displaying good awareness included being a male, visiting hospitals, and living in the West Bank and Jerusalem.
**Relevance**
Several misbeliefs and misconceptions regarding CRC causation were identified among the public in Palestine. Our results could potentially help in the development and implementation of appropriate educational interventions that aim to improve the public ability to distinguish evidence-based versus mythical causes of CRC.

There are several well-established risk factors for CRC, including lack of physical activity, having a diet rich in red meat and poor in fruits, vegetables, and fiber, in addition to smoking, alcohol consumption, aging, and family history.^3^ The ability to identify these risk factors may play a role in health behaviors that may reduce the individual's chance to develop CRC.^4^ A previous study from Palestine found that only 40% of participants displayed good awareness of CRC risk factors.^5^ However, the recognition of myths around CRC causation to be incorrect among the Palestinian population remains unknown. Moreover, efforts in behavior modification for CRC causes may be harder to implement if they have been influenced by beliefs that are not scientifically supported (ie, factitious causes). Factitious causes of CRC may deceive people and encourage them to place too much emphasis on unfounded risk factors rather than pursuing healthy, evidence-based lifestyle choices that may actually reduce their risk of developing CRC.^6^

Since no national CRC screening program exists in Palestine, recognition of warning signs is important for early presentation. However, awareness levels of CRC signs and symptoms, and risk factors in Palestine were found to be low in previous studies,^5,7^ highlighting a need to investigate abilities to recognize CRC causation myths to be incorrect among the Palestinian population. This could help in the development and implementation of appropriate educational interventions to improve public ability to distinguish evidence-based versus mythical causes of CRC.

Therefore, this study aimed to (1) examine awareness of myths around CRC causation to be incorrect in the Palestinian community, (2) compare this awareness between the Gaza Strip and the West Bank and Jerusalem (WBJ), and (3) and explore the factors associated with good awareness.

## MATERIALS AND METHODS

### Study Design, Setting, and Population

A national cross-sectional study was carried out in Palestine between July 2019 and March 2020. Adults make up approximately 52% (approximately 2.6 million) of the total population of Palestine (approximately 5.0 million).^5,8^ Adult Palestinians were the target population. Exclusion criteria were holding a nationality other than Palestinian, studying or working in health-related fields, visiting oncology departments at the time of data collection, and inability to complete the questionnaire.

### Sampling Methods

Most Palestinians receive their health care services at governmental hospitals and primary health care centers at no cost or low copayment. Convenience sampling was used to recruit eligible participants from governmental hospitals, primary health care centers, and public spaces in 11 governorates (seven in the WBJ and four in the Gaza Strip). Public spaces included parks, malls, marketplaces, mosques, transportation stations, and others. The recruitment from different governorates and geographic locations was intended to maximize the representativeness of the study cohort.^5,7,9-18^

### Questionnaire and Data Collection

A modified version of the Cancer Awareness Measure-Mythical Causes Scale (CAM-MYCS) was used for data collection.^19^ A back-to-back translation was done; two bilingual health care professionals translated CAM-MYCS from English to Arabic and another two bilingual health care professionals back-translated it from Arabic to English. These health care professionals had relevant expertise in coloproctology, public health, and survey design. The questionnaire was then assessed by five independent health care professionals and researchers for content validity. This was followed by conducting a pilot study (n = 25) to evaluate the clarity of the Arabic questionnaire. The questionnaires of the pilot study were not included in the final analysis. Internal consistency was evaluated using Cronbach's alpha, which reached an acceptable level of .84.

The questionnaire comprised two sections. The first section assessed the sociodemographic information of participants, while the second section assessed the prompt recognition of 13 myths related to CRC causation as being incorrect. Of the 13 myths, 12 were adapted from the original CAM-MYCS. Eating burnt food was added as the 13th myth because of it being a popular belief in the Palestinian community.^20^ To minimize the possibility of participants answering questions at random, the original questions of the CAM-MYCS with correct/incorrect/unsure responses were modified into questions scored on a 5-point Likert scale (ranging from 1 = strongly disagree to 5 = strongly agree). Answers with disagree or strongly disagree were considered correct; all other answers were considered incorrect.

Eligible participants were invited to a face-to-face interview to complete the questionnaire in the presence of a data collector. Data were collected using Kobo Toolbox (Cambridge, MA), a reliable and user-friendly application that can be used on smartphones.^21^

### Statistical Analysis

Participant characteristics were summarized using descriptive statistics, where categorical variables were described using frequencies and percentages, and continuous variables, which were non-normally distributed, were described using the median and IQR. Comparisons between baseline characteristics of participants from the Gaza Strip and those from the WBJ were performed using Kruskal-Wallis or Pearson's chi-square test if the characteristic was continuous or categorical, respectively.

The recommendations of the American Cancer Society state that people at average risk of developing CRC should start screening at age 45 years.^22^ Therefore, this cutoff was used to dichotomize the continuous variable of age into two categories: 18-44 years and ≥45 years. In addition, the minimum wage in Palestine is 1,450 New Israeli Shekel (NIS; about $450 in US dollars),^23^ therefore, this cutoff was used to dichotomize the continuous variable of monthly income into two categories: <1,450 NIS and ≥1,450 NIS.

The evaluated myths related to CRC causation were classified into food-related and food-unrelated. The recognition of each myth was described using frequencies and percentages, and comparisons between participants from the Gaza Strip versus those from the WBJ were performed using Pearson's chi-square test.

Multivariable logistic regression analyses were used to examine the association between participant characteristics and recognizing each myth related to CRC causation. The multivariable analyses adjusted for age group, sex, educational level, employment status, monthly income, place of residence, marital status, having a chronic disease, following a vegetarian diet, knowing someone with cancer, and site of data collection. This model was determined a priori on the basis of previous studies.^5-7,19,24-27^

The awareness level of the myths about CRC causation was assessed using a scoring system, which had also been used in previous studies.^5,7,12,14,16,18^ One point was given for each correctly recognized myth. The awareness level was determined based on the total score (ranging from 0 to 13), which was calculated and classified into three categories on the basis of the number of myths recognized: poor (0-4), fair (5-9), and good awareness (10-13). The awareness levels were compared between participants from the Gaza Strip versus those from the WBJ using Pearson's chi-square test. A multivariable logistic regression analysis was used to examine the association between participant characteristics and displaying good awareness. The same aforementioned multivariable model was used.

Missing data were hypothesized to be missed completely at random and thus, complete case analysis was used to handle them. Data were analyzed using Stata software version 17.0 (StataCorp, College Station, TX).

### Ethics Approval and Consent to Participate

This study was approved by the Helsinki Committee in the Gaza Strip, which is responsible for providing ethical approvals for health-related research studies. It was also approved by the Research Ethics Committee at the Islamic University of Gaza and The Human Resources Development department at the Ministry of Health. In addition, the appropriate local laws and regulations were followed in the execution of all study procedures. After receiving a thorough description of the goals of the study, all participants granted their consent to participate in the study. All participants were made aware that their participation was entirely voluntary and had no effect on the medical care they would receive. All the methods of the study were carried out in accordance with relevant local guidelines and regulations.

## RESULTS

### Participant Characteristics

Of the 5,254 potential participants approached, 4,877 agreed and completed the questionnaire (response rate = 92.3%). The final analysis included 4,623 questionnaires: 2,700 from the WBJ and 1,923 from the Gaza Strip. A total of 254 questionnaires were excluded: 44 did not meet the inclusion criteria and 210 had missing data. The median age (IQR) of all participants was 31.0 years (24.0-43.0), and 1,879 (40.6%) of them were males (Table 1). The WBJ participants were relatively higher in percentage of older age, greater monthly income, and chronic illnesses than the Gaza Strip participants.

**TABLE 1:**
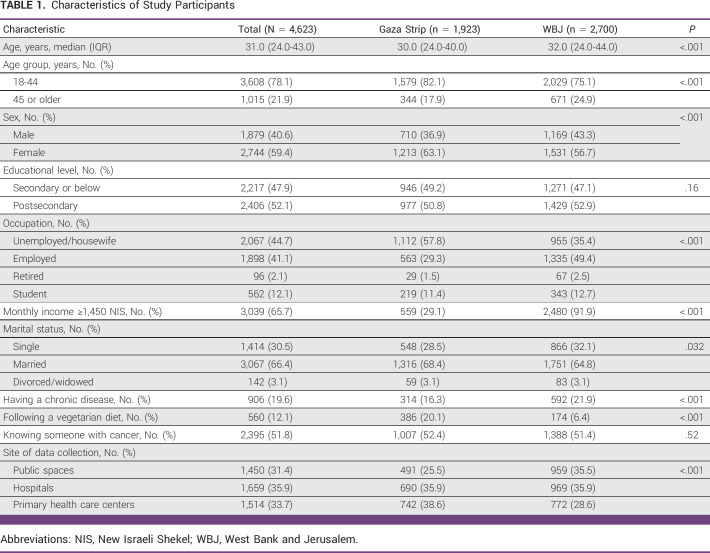
Characteristics of Study Participants

### Good Recognition of CRC Causation Myths and Its Associated Factors

**FIG 1:**
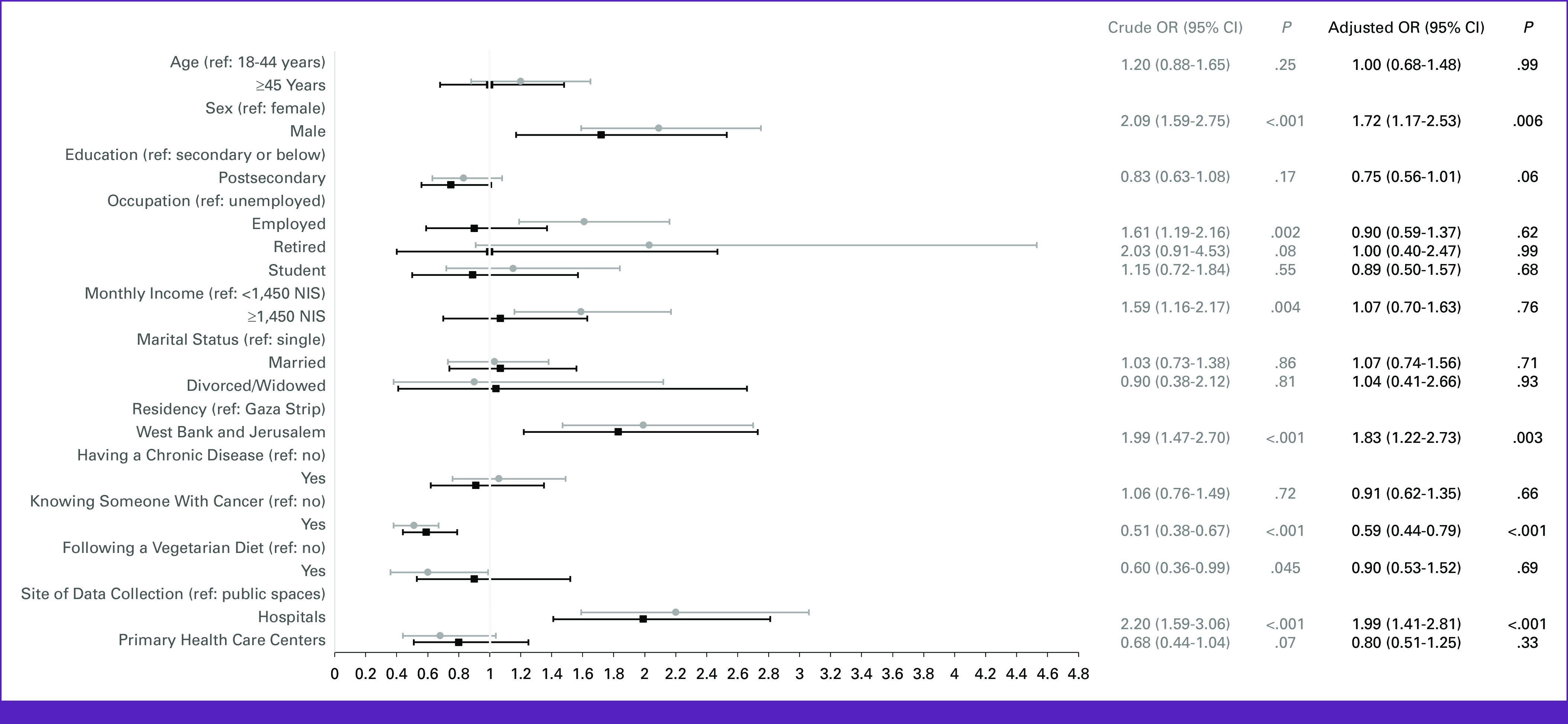
Forest plot summarizing the results of bivariable and multivariable logistic regression analyses examining factors associated with having good recognition of the mythical causes of colorectal cancer. NIS, New Israeli Shekel; OR, odds ratio; Ref, reference.

Only 219 participants (4.7%) demonstrated good awareness levels (ie, prompt recognition of more than nine of the 13 CRC mythical causes; Table 2). Participants from the WBJ were about twice more likely than those from the Gaza Strip to display good awareness (5.9% *v* 3.1%). On the multivariable analysis, male sex, living in the WBJ, and visiting a hospital were all associated with an increase in the likelihood of displaying good awareness (Fig 1). Conversely, knowing someone with cancer was associated with a decrease in the likelihood of displaying good awareness.

**TABLE 2:**
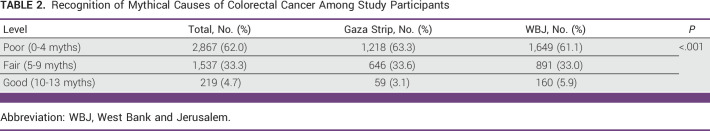
Recognition of Mythical Causes of Colorectal Cancer Among Study Participants

### Recognition of CRC Causation Myths to Be Incorrect

Overall, myths about CRC causation unrelated to food were more frequently recognized than those related to food. Eating burnt food was the most frequently recognized food-related myth (n = 1,157, 25.0%), followed by drinking from plastic bottles (n = 1,111, 24.0%; Table 3). Eating food containing additives was the least frequently recognized food-related myth (ie, most participants incorrectly identified it as a risk factor; n = 456, 9.8%). Having a physical trauma was the most recognized food-unrelated myth (n = 2,752, 59.5%), whereas the least recognized was feeling stressed (n = 1,449, 31.3%).

**TABLE 3:**
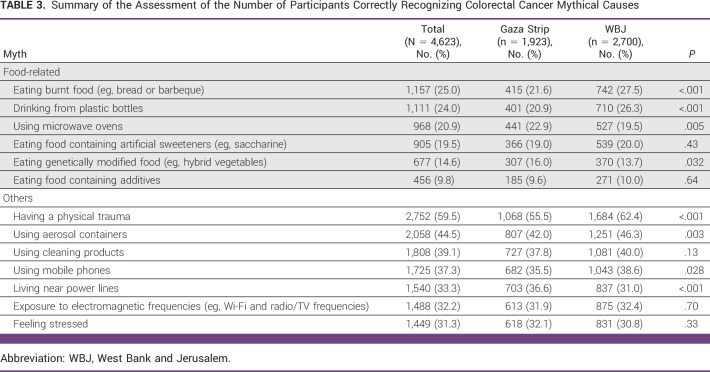
Summary of the Assessment of the Number of Participants Correctly Recognizing Colorectal Cancer Mythical Causes

### Association Between Participant Characteristics and Recognizing CRC Food-Related Mythical Causes

Male participants were more likely than female participants to recognize all CRC food-related myths except eating genetically modified food, where no associated difference was found (Table 4). Similarly, vegetarian participants were more likely than nonvegetarian participants to recognize all CRC food-related myths except eating food containing artificial sweeteners, where no associated difference was observed. In addition, participants recruited from hospitals were more likely than those recruited from public spaces to identify half of the CRC food-related myths. By contrast, participants who knew someone with cancer were less likely than participants who did not to recognize all CRC food-related myths correctly, except eating burnt food, where no associated difference was found. Education level was not associated with recognizing CRC food-related mythical causes correctly except for eating burnt food, where participants with higher education (ie, postsecondary) were less likely to recognize it (odds ratio, 0.78 [95% CI, 0.68 to 0.91]).

**TABLE 4:**
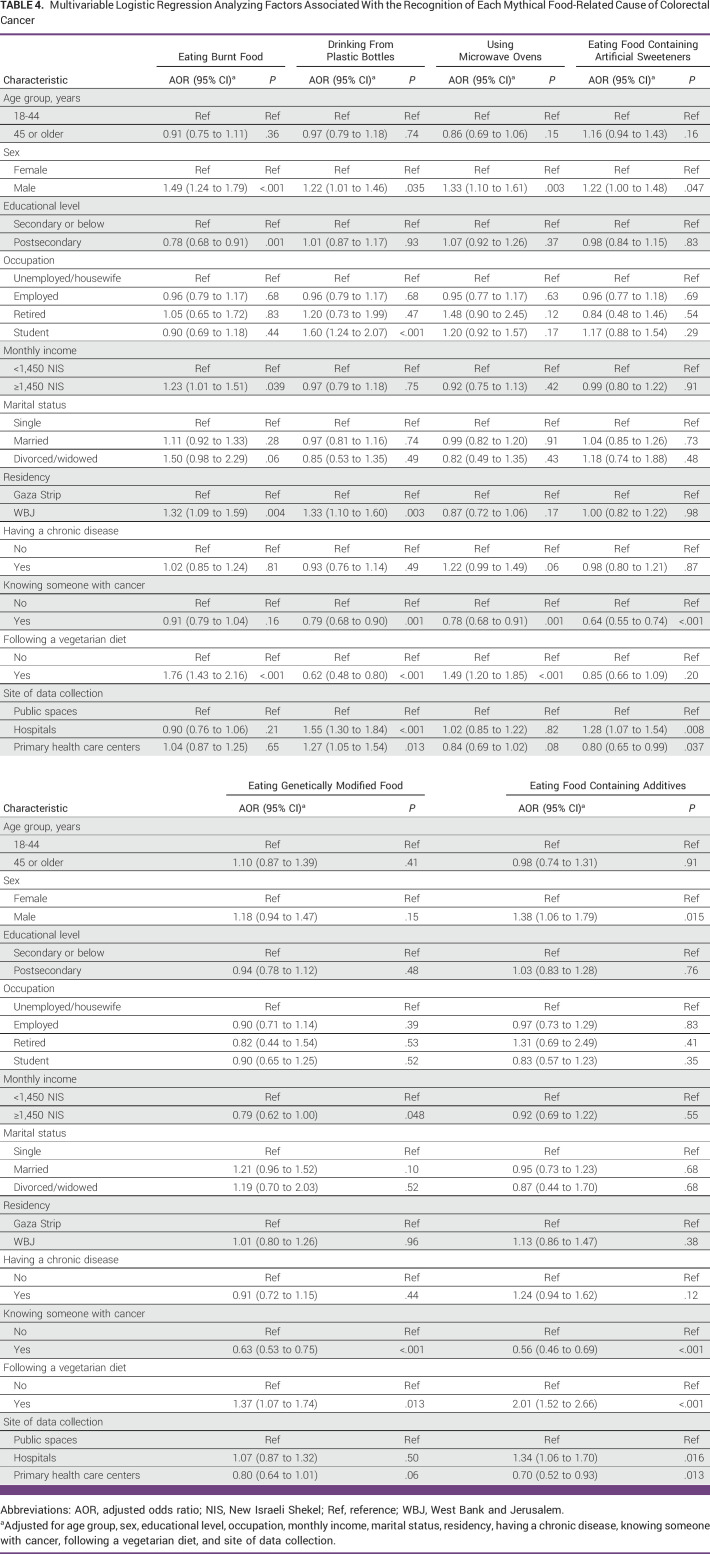
Multivariable Logistic Regression Analyzing Factors Associated With the Recognition of Each Mythical Food-Related Cause of Colorectal Cancer

### Association Between Participant Characteristics and Recognizing CRC Food-Unrelated Mythical Causes

Male participants were more likely than female participants to correctly recognize all other CRC mythical causes except having a physical trauma and using aerosol containers, where no associated differences were found (Table 5). Similarly, participants recruited from hospitals were more likely than those recruited from public spaces to correctly identify all other CRC mythical causes except having a physical trauma and living near power lines, where no associated differences were found. In addition, participants recruited from primary health care centers were more likely than those recruited from public spaces to correctly recognize four of seven CRC food-unrelated mythical causes.

**TABLE 5:**
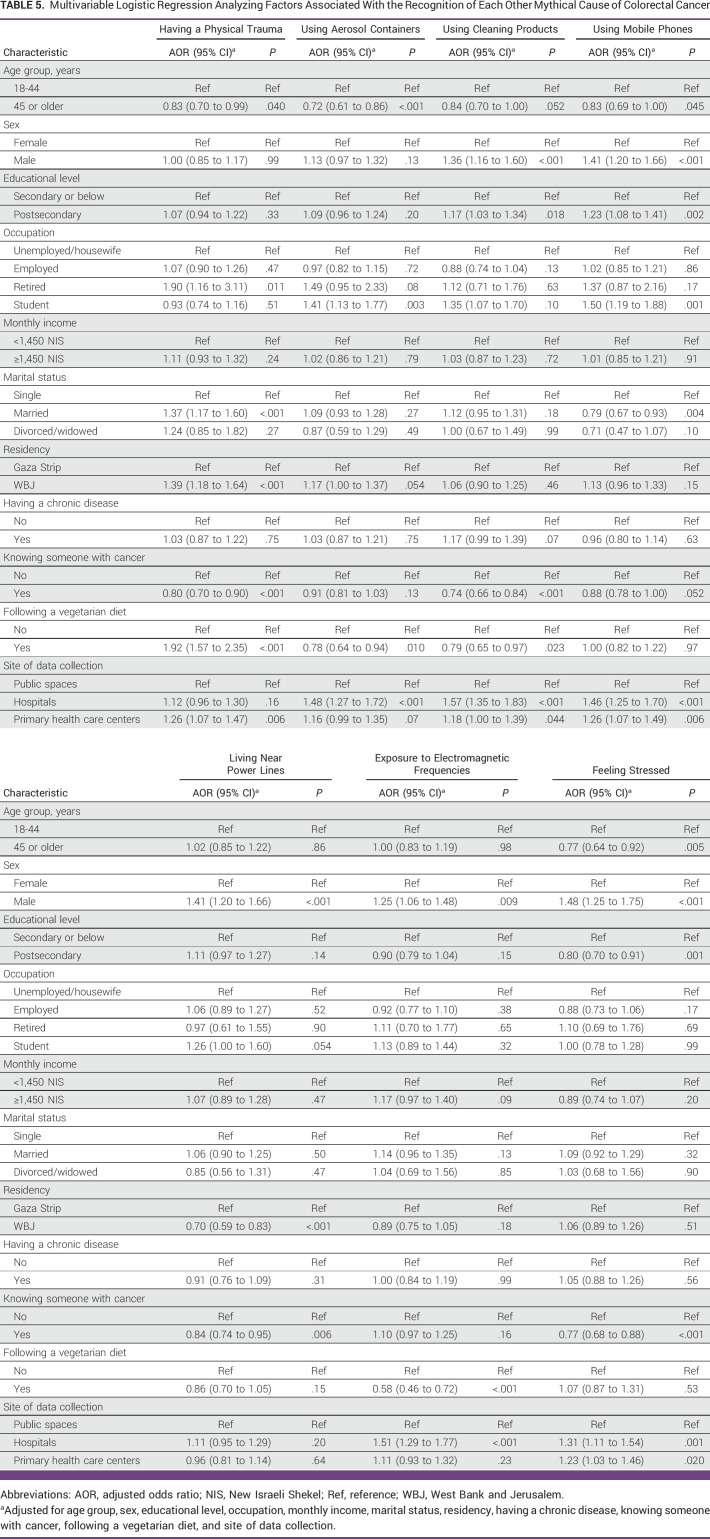
Multivariable Logistic Regression Analyzing Factors Associated With the Recognition of Each Other Mythical Cause of Colorectal Cancer

By contrast, older participants (age 45 years and older) were less likely than younger participants to recognize four of the seven CRC food-unrelated mythical causes. In addition, participants who knew someone with cancer were less likely than participants who did not to recognize four of the seven CRC food-unrelated mythical causes.

## DISCUSSION

Myths around CRC causation unrelated to food were more frequently recognized to be incorrect than those related to food. Only 4.7% of the participants demonstrated good awareness of CRC mythical causes. Being a male, living in the WBJ, and visiting hospitals were all associated with an increase in the likelihood of displaying good awareness. Conversely, knowing someone with cancer was associated with a decrease in the likelihood of displaying good recognition.

In the United Kingdom, one study demonstrated that up to 50% of patients diagnosed with CRC demonstrated clear risk factors in lifestyle behaviors.^28^ Furthermore, in the United States, another study showed that up to 40% of new cancer cases could be prevented by identifying risk factors and avoiding them (ie, implementing a risk-reducing lifestyle).^29^ In the United Kingdom, it is estimated that more than one million cancer cases could be prevented by following healthy lifestyles.^28^ Correct recognition of CRC risk factors could facilitate adoption of healthier behaviors and increase the likelihood of participation in screening programs and thus, improve prevention and/or early detection of CRC.^30^ Nevertheless, myths and misconceptions about different cancers contribute to people avoiding these healthy lifestyles and risk-reduction measures.^5,24,31^ For instance, the adoption of alternative medicines, diets, and lifestyle choices may be affected by knowledge of the various causes of cancer (both genuine and also factitious causes).^32^ In addition, false beliefs of cancer can affect the tendency of patients to visit a doctor depending on the site of the tumor in the body.^6^ Increasing the public recognition level of the factitious causes of CRC might help in targeting efforts toward the public focusing on the evidence-based, modifiable risk factors, which may help reduce the associated morbidity and mortality.

Similar to this study, recognition of CRC myths was poor in a study from Pakistan.^33^ However, other studies conducted in the United Kingdom, United States, and Spain showed better results.^24,27,34^ This might indicate that recognition of CRC myths could be better in high-income countries, compared with low- and middle-income countries, possibly because of public education interventions. Studies in high-income countries have shown the effectiveness of educational campaigns in increasing public awareness of colorectal and abdominal cancers.^35,36^

The poor recognition of CRC factitious causes in Palestine could be related to misinformation on social media in Palestine. The number of Internet users in Palestine was over 3.7 million in 2022 and is increasing each year.^37^ There are concerns that Internet, especially social media, can be a source of false medical information. Loeb et al^38^ investigated the content of social media on YouTube containing medical information about bladder cancer, where approximately 70% were classified as either bad or moderate quality. In addition, Johnson et al^39^ assessed medical information in the most popular articles regarding lung, prostate, colorectal, and breast cancers in several social media websites (eg, Facebook, Twitter, and Pinterest), where nearly 30% of the information found was false and 77% could lead to harm of patients, misuse of medications, or endorsement of unproven practices. More attention should be given to the role of social media when trying to increase public awareness, and individuals must be encouraged to validate information sources from the Internet before endorsing them or taking actions based on them.^40^ However, verifying information can be more challenging in low-income settings like Palestine, where there is a dearth of trustworthy information in public discourse and national educational interventions are lacking.

Anxiety and post-traumatic stress disorder are the most common psychiatric disorders in Palestine.^41^ However, feeling stressed was the least correctly recognized food-unrelated cause of CRC (31.3%). This recognition level is lower than that of a study that assessed recognition of myths among 22 different countries, where the highest recognition rates for stress to be a mythical cancer cause were found in Bulgaria.^42^ However, our results showed better awareness than some previous studies conducted in countries such as Ireland (92% of respondents endorsed stress as a strong risk factor of cancer)^43^ and Australia (78% of participants considered stress as a risk factor for cancer).^44^ Therefore, there appears to be a common misconception in Palestine about the relationship between stress and the development of cancer, which could be targeted in educational interventions on mental health.

Consistent with a previous study conducted in the United Kingdom,^24^ male participants in this study demonstrated better abilities to recognize cancer causation myths to be incorrect compared with female participants. However, in a previous study from Palestine,^5^ female participants showed better awareness levels of CRC risk factors. Furthermore, Carnahan et al^45^ found that males diagnosed with CRC had a lower level of awareness regarding the different screening methods of CRC. It is possible that female participants have greater concerns than males about health issues, making them more aware of risk factors and screening opportunities while also seeing fictitious causes of CRC as genuine hazards.^24,45^

Interestingly, vegetarians correctly recognized the majority of CRC food-related mythical causes. Vegetarians in general tend to be more knowledgeable about nutrition and the different ingredients in food. They also have a higher awareness of the dietary causes of CRC, as well as having lower mortality rates from different diseases including cancer because of their balanced diet.^31,46^ This underlines the potential impact educational interventions could have on cancer mortality if adoption of healthier lifestyle choices were attained.

Surprisingly, like this study, a Spanish study showed poorer abilities to recognize CRC myths as incorrect among people who knew someone with cancer.^27^ This might be due to a heightened receptivity to messages around CRC causation among people who know someone with cancer.^47,48^ Furthermore, the scarcity of evidence-based public information campaigns in low-income settings, such as Palestine, may lead people to search for information from less reliable sources, including social media^40,49^ and, thus, expose people to non–evidence-based messages around CRC without exposure to balanced evidence-based information. Thus, people who know someone with cancer might have more exposure to non–evidence-based information as they seek knowledge and are more receptive to messages around CRC, making them more vulnerable to misinformation.^47,48,50^ In Middle Eastern countries, families play an important role in medical and lifestyle decisions made by patients, which can affect treatment options and opportunities for care.^6,51^ Families are often the main carers for patients diagnosed with cancer in the Middle East and also take responsibility for treatment,^6^ compared with Western countries, where care and treatment choices are more strongly dependent on patient preferences and where hospices or nursing homes are more acceptable and available.^52^ One study in Taiwan showed that older Taiwanese residents at long-term care institutions believe firmly that they must contact their families before making any decisions connected to end-of-life care, in contrast to older Europeans and Americans,^53,54^ while another study showed that family members participated in decision making in <50% of all non-Hispanic White patients with cancer, with percentages being higher for Asian Americans and Spanish-speaking Latinos.^55^ Therefore, while raising awareness of different cancers, there should be more consideration of the impact that family members and friends have on lifestyle choices and behaviors.

Another important consideration for future educational interventions could be the interaction between health care professionals and patients. In this study, participants recruited from hospitals were more likely than those recruited from public spaces to display good awareness and to identify most CRC causation myths. This may indicate the importance of the health care professionals' role in spreading the awareness of CRC risk factors and refuting unfounded causes. This is supported by findings of a previous national study from Palestine that measured the awareness of CRC risk factors and found that individuals recruited from hospitals were more likely to identify most CRC risk factors.^5^ In addition, previous studies have shown that good communication between health care workers and patients can affect their participation in screening programs for CRC.^7,56^

Convenience sampling was used to recruit participants, which might affect the generalizability of the results. However, the large sample size and sampling from different geographical locations in Palestine could help in mitigating this limitation. Moreover, the exclusion of participants with medical background and presumably higher awareness may have affected our results. However, this was intended to increase the relevance of the study to the Palestinian public. A further limitation could be that some sociodemographic data, namely having a chronic disease, following a vegetarian diet, and knowing someone with cancer, were self-reported by study participants, which may not be the best way to collect data for these factors.

In conclusion, participants of this study demonstrated inadequate knowledge of factors not proven to be causing CRC, with only 4.7% displaying good awareness. Food-related myths around CRC causation were less frequently recognized to be incorrect than those unrelated to food. Factors associated with higher likelihood of displaying good awareness included male sex, living in the WBJ, and visiting hospitals. Conversely, knowing someone with cancer was associated with lower likelihood of displaying good awareness. The results of this study highlight the need for educational campaigns and interventions to increase awareness of CRC causation myths.

## Data Availability

The data set used and analyzed during the current study is available from the corresponding author on reasonable request.
